# Effect of long-term inorganic nitrate administration on myocardial ischemia-reperfusion injury in ovariectomized rats

**DOI:** 10.3389/fphar.2024.1369379

**Published:** 2024-03-27

**Authors:** Sajad Jeddi, Nasibeh Yousefzadeh, Maryam Zarkesh, Khosrow Kashfi, Asghar Ghasemi

**Affiliations:** ^1^ Endocrine Physiology Research Center, Research Institute for Endocrine Sciences, Shahid Beheshti University of Medical Sciences, Tehran, Iran; ^2^ Cellular and Molecular Endocrine Research Center, Research Institute for Endocrine Sciences, Shahid Beheshti University of Medical Sciences, Tehran, Iran; ^3^ Department of Molecular, Cellular, and Biomedical Sciences, Sophie Davis School of Biomedical Education, City University of New York School of Medicine, New York, NY, United States

**Keywords:** ovariectomy, menopause, nitric oxide, nitrate, cardiac function, female rats

## Abstract

**Introduction:** Menopause is associated with reduced nitric oxide (NO) bioavailability and lower tolerance against myocardial ischemia-reperfusion (IR) injury. This study investigated whether long-term nitrate administration provides resistance against myocardial IR injury in ovariectomized (OVX) rats.

**Method:** After ovariectomy, female rats were assigned to the OVX and the OVX + nitrate groups (*n* = 14/group); the latter group consumed nitrate (100 mg/L) for 9 months. At month 9, each group was divided into two subgroups (*n* = 7/subgroup), of which one subgroup was exposed to myocardial IR (IR^+^ hearts) and the other was not exposed (IR^−^ hearts). The hearts of rats were isolated, and NO metabolite (NOx), oxidative stress indices, and mRNA expressions of endothelial (eNOS), inducible (iNOS), and neuronal (nNOS) NO synthases, as well as markers of apoptosis, were measured in the IR^−^ and IR^+^ hearts. In the IR^+^ hearts, cardiac function indices (CFI) and the infarct size were also measured.

**Results:** Nitrate increased catalase activity (97%) and eNOS expression (2.94-fold) in the IR^−^ hearts. In the IR^+^ hearts, nitrate reduced left ventricular (LV) end-diastolic pressure (11.6%) and infarct size (26.2%) and increased recovery of LV developed pressure (44.0%) and peak rate of positive (28.9%) and negative (15.4%) changes in LV pressure. In addition, in the IR^+^ hearts, nitrate increased eNOS and B-cell lymphoma-2 (Bcl-2) as well as decreased iNOS, Bcl-2 associated X protein (Bax), caspase-3, caspase-8, caspase-9, and tumor necrosis factor-α (TNF-α) expression. Nitrate increased total antioxidant capacity (TAC) and catalase (CAT) activity and decreased malondialdehyde (MDA) levels at month nine in serum and IR^+^ hearts.

**Conclusion:** The favorable effects of nitrate against IR injury were associated with higher eNOS and Bcl-2 expression, CAT activity, TAC, and lower iNOS, Bax, caspase-3, caspase-8, caspase-9 and TNF-α expression, and MDA in the heart tissue. Nitrate preconditioning alleviated IR-induced myocardial injury in OVX rats; this effect was associated with eNOS upregulation before IR and the blunting of OVX-induced eNOS downregulation, iNOS upregulation, apoptosis, and oxidative stress in heart tissue after IR.

## 1 Introduction

Ischemic heart disease (IHD) is a leading cause of death globally (NCD Countdown collaborators, 2020) and is responsible for 9.14 million deaths in 2019, registering a 60% increase from 1990 (Safiri et al., 2022). The risk of IHD is 30% for premenopausal women (Lloyd-Jones et al., 1999), which increases after menopause by 200%–400% (Costello et al., 2017). Surgical menopause is a leading cause of early menopause in women (Faubion et al., 2015), and compared to natural menopause, it increases the risk of IHD by 50% (Muka et al., 2016). Hormone replacement therapy (HRT) is the first-line treatment in women with surgical menopause; however, the frequency of HRT decreased from ∼90% before 2002 to 10%–50% in 2020 (Marjoribanks et al., 2012; Sarrel et al., 2016; Garg et al., 2020) due to its possible side effects.

Reduced tolerance against myocardial ischemia-reperfusion (IR) injury and increased infarct size by 45%–75% has been reported in ovariectomized (OVX) rats following 2 (Kaya and Agan, 2022), 6 (Garvin et al., 2017), 12 (Kumar et al., 2021), and 44 (Yousefzadeh and Jeddi, 2023) weeks after ovariectomy. We previously reported that these effects of OVX in heart tissue are at least in part associated with reduced nitric oxide (NO) levels, reduced endothelial NO synthase (eNOS) expression, and increased inducible NOS (iNOS) expression (Yousefzadeh and Jeddi, 2023). In addition, in heart tissue of OVX rats, increased activation of Fas-receptor-dependent extrinsic pathway of apoptosis, including Fas ligand, caspase-3, caspase-8 and tumor necrosis factor-α (TNF-α), as well as mitochondrion-dependent intrinsic pathway of apoptosis, including Bcl-2 associated X protein (Bax), B-cell lymphoma 2 (Bcl-2), cytochrome c, caspase-3, and caspase-9 has been reported (Lee et al., 2008). Dietary nitrate and nitrite can boost NO in NOS-disrupted conditions (Lundberg et al., 2015) and reduce IHD risk by 21% (Bryan, 2017). In support, in male mice, a low-nitrite diet for 1 week exacerbates myocardial IR injury (Bryan et al., 2007), and in humans, consuming one fruit/vegetable serving per day as a source of inorganic nitrate reduces the risk of coronary heart disease by 4% (Joshipura et al., 2001). In addition, nitrate may also have estradiol-like effects in heart tissue; following conversion from nitrate, nitrite directly binds to the ligand-binding domain of estrogen receptor alpha (ERα) (Veselik et al., 2008), while NO S-nitrosylates ERα (Garbán et al., 2005). These actions mediate and restore the favorable effects of estradiol in heart tissue (Caulin-Glaser et al., 1997).

The protective effects of nitrate against myocardial IR have been studied in the short-term (only 1 week before ischemia) and in male rodents (Bryan et al., 2007; Salloum et al., 2015). Only two studies from our laboratory addressed the favorable long-term effects of nitrate against myocardial IR for 2 (Jeddi et al., 2016) and 9 (Yassaghi et al., 2023b) months in male diabetic (Jeddi et al., 2016) and normal female (Yassaghi et al., 2023b) rats. Time-dependent effects of nitrate in OVX rats have been reported previously; for example, glucose-lowering and anti-osteoporotic effects of nitrate have been observed only after 6 months of intervention (Yousefzadeh et al., 2022b; Yousefzadeh et al., 2022c). In addition, compared to males, following ischemia, females have reduced myocardial IR injury (Besík et al., 2007) and infarct size (Bae and Zhang, 2005), as well as higher eNOS and neural NOS (nNOS) expression (Sun et al., 2006) and lower apoptosis (Chen et al., 2013; Medzikovic et al., 2023) in heart tissue. Therefore, the current study was designed to investigate whether the long-term (9 months) nitrate administration provides resistance against myocardial IR injury in OVX rats.

## 2 Methods

### 2.1 Ethical approval

Following the principles of the 3Rs (Replacement, Reduction, and Refinement), to minimize the number of rats used in the study, isolated hearts and serum were obtained from an earlier study in which we reported that long-term nitrate administration improved carbohydrate metabolism in OVX rats (Yousefzadeh et al., 2022c). The present study focused on the effect of nitrate administration on myocardial IR injury in OVX rats. All experiments in this study were conducted according to the published guidelines for the care and use of laboratory animals in Iran (Ahmadi-Noorbakhsh et al., 2021) and were reported following ARRIVE guidelines (Percie du Sert et al., 2020). The Research Institute for Endocrine Sciences ethics committee, affiliated with Shahid Beheshti University of Medical Sciences, approved all experimental procedures of the present study (Ethic Code: IR. SBMU.ENDOCRINE.REC.1402.084).

### 2.2 Study protocol

**FIGURE 1:**
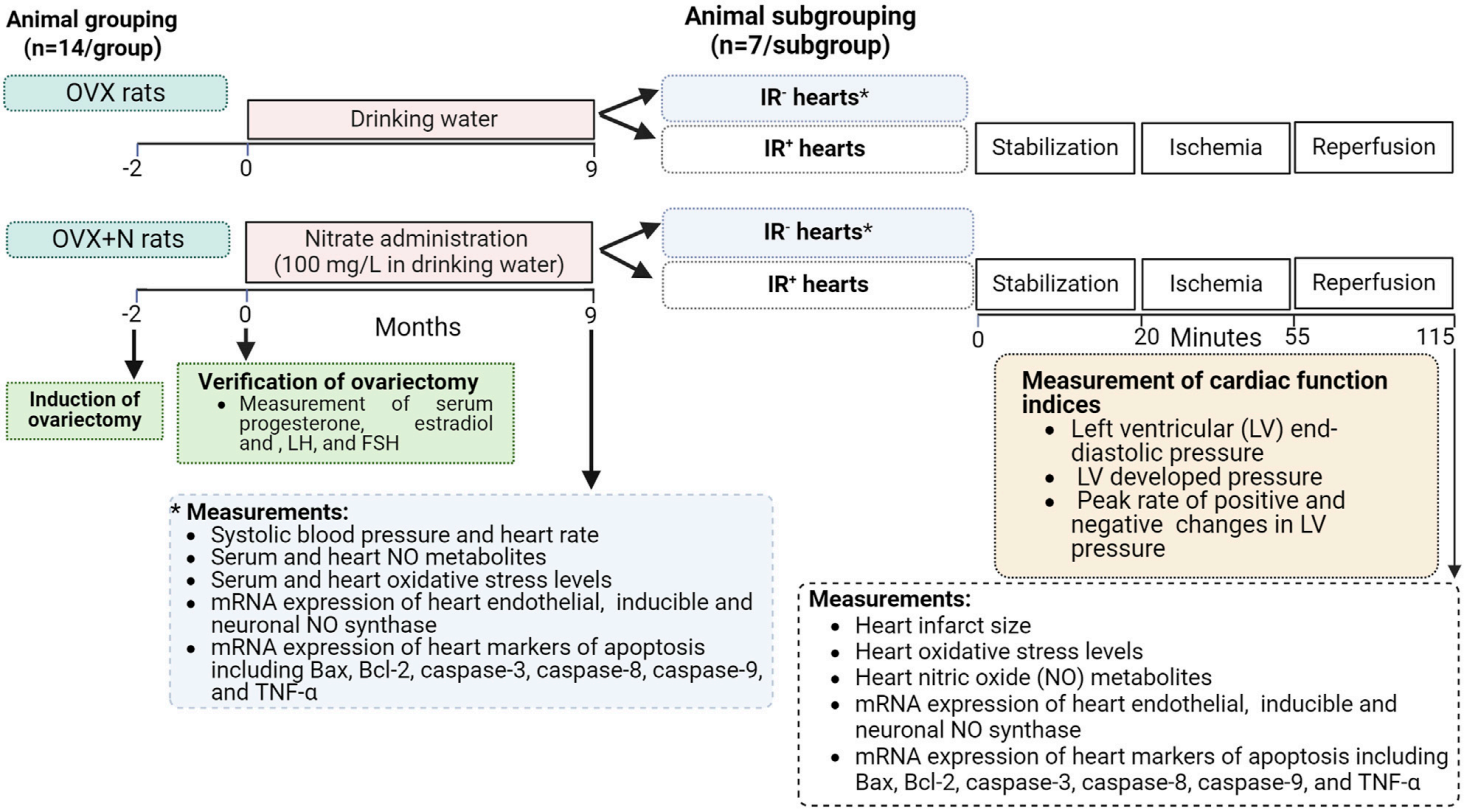
Study protocol. Bax, Bcl-2 associated X protein; Bcl-2, B-cell lymphoma-2; FSH, follicle-stimulating hormone; IR^+^, exposed to ischemia-reperfusion; IR^−^, not-exposed to ischemia-reperfusion; LH, luteinizing hormone; OVX, ovariectomized; OVX + N, OVX + nitrate; TNF-α, tumor necrosis factor-α.

This experimental interventional study was conducted in OVX rats (6-month-old, 200–215 g) maintained under standardized conditions with unrestricted access to a standard diet and drinking water. The study protocol is presented in Figure 1. A total of 28 rats were randomly assigned to two groups (*n* = 14/group): the OVX group, which received tap water, and the OVX + nitrate (OVX + N) group, which consumed sodium nitrate (100 mg/L) in tap water for 9 months. The period of study (9 months) was chosen for two reasons. First, previous studies have shown that long-term periods (>6 months) are needed to observe the adverse effects of ovariectomy on heart function (Shinmura et al., 2008; Tang et al., 2016; Yousefzadeh et al., 2022a). Second, long-term administration is necessary to observe the potential protective effects of nitrates in OVX rats, including glucose-lowering and anti-osteoporotic effects, as indicated by previous studies (Yousefzadeh et al., 2022b; Yousefzadeh et al., 2022c). At the end of the intervention, both groups were further subdivided into two subgroups (*n* = 7/subgroup), with the hearts of one subgroup subjected to IR (IR^+^ hearts) and the other subgroup left untreated (IR^−^ hearts).

Body weight, systolic blood pressure (SBP), and heart rate (HR) were measured at the end of the study (month 9). Blood samples were taken from the tail tips of all rats under isoflurane inhalation anesthesia at month 9, centrifuged (3,000 g for 10 min), and sera were used to measure progesterone, estradiol, follicle-stimulating (FSH), and luteinizing hormone (LH) as well as oxidative stress indices. In addition, the hearts of all rats were isolated under sodium pentobarbital anesthesia (60 mg/kg, intraperitoneal injection). Nitric oxide metabolite (NOx) concentration, oxidative stress indices, and mRNA expressions of eNOS, iNOS, and nNOS, as well as Bax, Bcl-2, caspase-3, caspase-8, and caspase-9, as well as TNF-α were measured in IR^−^ and IR^+^ hearts. In IR^+^ hearts, cardiac function indices (CFI) and infarct size were also measured in addition to these parameters.

### 2.3 Measurement of parameters for verification of ovariectomy

Following an overnight fast, rats (200–215 g body weight) were anesthetized with sodium pentobarbital (60 mg/kg, intraperitoneally), and the ovaries were removed via a dorsolateral skin incision method, which has been described in detail in our previous publication (Yousefzadeh et al., 2020). To confirm the ovariectomy, serum progesterone, estradiol, FSH, and LH levels were measured 2 months after ovariectomy at month 0. The sensitivities of the assay kits were 0.1 ng/mL for progesterone, 10 pg/mL for estradiol, 0.7 mIU/mL for FSH, and 0.15 mIU/mL for LH. The kits for measuring progesterone and estradiol were obtained from Diagnostics Biochem (Ontario, Canada), and those for measuring FSH and LH were obtained from Cusabio Biotech (Wuhan, China). The intra-assay coefficients of variation (CVs) were less than 7% for all assays.

### 2.4 Measurement of SBP and HR

At month 9, rats were individually placed in a restrainer, and the noninvasive tail-cuff method (AD Instruments, MLT125R, Australia) was used to assess *in vivo* SBP and HR. For each rat, SBP and HR values were averaged from three consecutive measurements.

### 2.5 Isolated heart and assessment of CFI

The isolation of the heart and assessment of CFI were described in detail in our previous publication (Yassaghi et al., 2023b). In brief, at month 9, OVX rats were anesthetized with sodium pentobarbital (60 mg/kg, intraperitoneal injection), and hearts were surgically removed. The isolated hearts were immediately immersed in ice-cold, carbogenated Krebs-Henseleit solution (composition in mM: 118.6 NaCl; 4.7 KCl; 2.5 CaCl_2_; 1.6 MgSO_4_; 1.2 KH_2_PO_4_; 25 NaHCO_3_; 11.1 glucose - all from Merck, Darmstadt, Germany) to minimize ischemic damage and after that cannulated in Langendorff apparatus. Aortic cannulation enabled retrograde Langendorff perfusion in isolated hearts. After being inserted into the LV, a deflated LV balloon was inflated to 5–10 mm Hg and connected to a pressure transducer (MLT844-Sweden) via a water-filled PE-50 tube. The transducer was coupled with a PowerLab system (AD Instruments, ML866, Australia) to record CFI parameters. CFI included left ventricular end-diastolic pressure (LVEDP), LV systolic pressure (LVSP), LV developed pressure (LVDP, obtained by subtracting the LVEDP from the LVSP), and peak rate of positive (+dp/dt) and negative (-dp/dt) changes in LV pressure. CFI was recorded during stabilization (20 min), global ischemia (35 min), and reperfusion (60 min) periods. After a 20-min stabilization, hearts underwent 35 min of no-flow global ischemia, induced by interrupting Krebs-Henseleit perfusion to the cannulated isolated heart and confirmed by the cessation of coronary effluent. Following the IR period, the hearts were removed from the Langendorff apparatus and stored at −80°C for future examinations.

### 2.6 Measurement of heart NO metabolites

At the end of the study, IR^−^ hearts and IR^+^ hearts were homogenized in phosphate-buffered saline (500 µL) and then centrifuged (10 min at 10,000 g); NOx and nitrite in all heart homogenates were then measured by the modified Griess method (Miranda et al., 2001) that reported previously in detail (Yousefzadeh et al., 2023). In brief, for deproteinization, zinc sulfate (10 μL, 15 mg/mL) and NaOH (10 μL, 3.72 M) were added to each sample (100 mg of tissue in 500 µL phosphate-buffered saline), centrifuged at 10,000 g for 10 min, and supernatants were used for NOx measurements. To measure NOx concentrations, nitrate was converted to nitrite by adding vanadium trichloride (8 mg/mL in 1 M HCl), after which N-(1-naphthyl) ethylenediamine (0.1% in ddH_2_O) and sulfanilamide (2% in 5% HCl) were added, and samples were incubated for 30 min at 37°C. The optical density was read at 540 nm. Nitrite was measured similarly, except that 1 M HCl was added to the samples to replace vanadium trichloride. The nitrate concentration in heart samples was determined by subtracting nitrite from NOx concentrations. The Bradford method was used to measure protein concentration in the samples (Bradford, 1976). NOx and nitrite levels are expressed as per mg protein, and intra-assay CVs of NOx and nitrite in heart tissue were less than 4%.

### 2.7 Measurement of infarct size

After the IR period, the triphenyl tetrazolium chloride (TTC) method was used to assess infarct size in isolated IR^+^ hearts. In brief, the iced hearts were meticulously sliced into thin segments, incubated in 1% TTC (in phosphate buffer solution) at 37°C for 10 min, and embedded in 10% formalin for 24 h (Ghanbari et al., 2015). This procedure vividly differentiated viable (red-stained) areas from necrotic (gray-stained) regions. The sliced heart sections were photographed, analyzed using ImageJ software, and represented as a percentage of the total area.

### 2.8 Measurement of mRNA expression

The sequences of the primers are provided in Table 1. The detailed protocols for RNA extraction, cDNA synthesis, and amplification have been previously described (Yousefzadeh et al., 2023). For RNA extraction, the RNX-Plus solution kit (Cinagen Co., Tehran, Iran) was used. For cDNA synthesis, the SMOBiO Technology cDNA synthesis kit (Taiwan) was used. To amplify cDNA, the Ampliqon SYBR Green Master Mix (Ampliqon Company, Denmark) was utilized using a real-time PCR machine (Rotor-Gene 6000; Corbett Life Science, Australia).

**TABLE 1:** Sequences of primers for target genes.

Name	Gene bank accession no	Sequence (5´→3´)	Size of product (bp)
Neuronal NOS	NM_052799	F: AAT​CTC​AGG​TCG​GCC​ATC​AC	126
		R: ATC​CCC​CAA​GGT​AGA​GCC​AT	
Endothelial NOS	NM_021838.2	F: TGA​CCC​TCA​CCG​ATA​CAA​CA	100
		R: CGG​GTG​TCT​AGA​TCC​ATG​C	
Inducible NOS	NM_012611	F: TGG​CCT​CCC​TCT​GGA​AAG​A	93
		R: GGT​GGT​CCA​TGA​TGG​TCA​CAT	
Bax	NM_017059.2	F: ACTAAAGTG CCCGAGCTGATC	141
		R: CAC​TGT​CTG​CCA​TGT​GGG​G	
Bcl-2	NM_016993.2	F: CGGGAGAACAGGGTATGA	149
		R: CAG​GCT​GGA​AGG​AGA​AGA​T	
Caspase-3	NM_012922.2	F: GGT​TCA​TCC​AGT​CAC​TTT​GC	77
		R: CCA​GGG​AGA​AGG​ACT​CAA​AT	
Caspase-8	NM_022277.1	F: TGG​GAA​GGA​TCG​ACG​ATT​AC	79
		R: CCA​CAT​GTC​CTG​CAT​TTT​GA	
Caspase-9	NM_031632.1	F: GAA​CGA​CCT​GAC​TGC​TAA​GA	72
		R: GGC​CTG​GCA​GCC​ATG​AGA​GA	
TNF-α	NM_012675.2	F: ACA​CAC​GAG​ACG​CTG​AAG​TA	195
		R: GGA​ACA​GTC​TGG​GAA​GCT​CT	
ß-actin	NM_031144.3	F: CGT​CCA​CCT​GCT​AGT​ACA​AC	100
		R: CGA​CGA​CTA​GCT​CAG​CGA​TA	

Bax, Bcl-2, associated X protein; Bcl-2, B-cell lymphoma-2; NOS, nitric oxide synthase; TNF-α, tumor necrosis factor-α.

### 2.9 Measurement of heart oxidative stress indices

Details on the measurement of serum and tissue oxidative stress indices, including total antioxidant capacity (TAC), catalase (CAT) activity, and malondialdehyde (MDA) levels, have been previously reported (Yousefzadeh et al., 2022b). In brief, TAC was measured by the ferric-reducing antioxidant power (FRAP) assay, which involves incubating the sample with a ferric-tripyridyltriazine complex and measuring the reduction of the complex to the ferrous form. CAT activity was measured by the Hadwan method, which involves incubating the sample with hydrogen peroxide and measuring the reduction of dichromate acetic acid. MDA levels were measured by the Satoh method, which involves extracting the MDA from the sample, converting it to a pink-colored complex, and measuring the absorbance of the complex. Intra-assay CVs for TAC, CAT, and MDA were less than 5%.

### 2.10 Statistical analyses

Data analysis was performed using GraphPad Prism software. Mean ± SEM was used for all parameters except mRNA expressions, which were presented as relative fold changes. The Student’s t-test compared parameters, including, SBP, HR, infarct size, and oxidative stress indices. The paired *t*-test compared body weight, serum progesterone and estradiol, FSH, and LH concentrations. One-way ANOVA followed by the Bonferroni *post hoc* test was used to compare nitrite, nitrate, and NOx levels. To compare CFI in IR^+^ hearts at different time points, two-way mixed (between-within) ANOVA was applied, followed by the Bonferroni post-hoc test. Relative gene expression was calculated using REST software. *p*-values <0.05 were considered statistically significant.

## 3 Results

### 3.1 Verification of ovariectomy

Compared to before ovariectomy (month −2), 2 months after ovariectomy (month 0), OVX rats had a lower concentration of progesterone by 74% (*p* < 0.001) and estradiol by 64% (*p* = 0.005) and a higher concentration of FSH by 6-fold (*p* = 0.011) and LH by 28-fold (*p* = 0.014) in serum (Table 2). In addition, OVX rats had higher body weight at month 0 by 21% (250.1 ± 5.4 vs 206.0 ± 2.3, *p* < 0.001). All of these changes confirm the inducing of the OVX rat model at month 0 before nitrate intervention.

**TABLE 2:** Effect of ovariectomy on serum estradiol, progesterone, luteinizing hormone, and follicle-stimulating hormone in rats.

Parameter	Before ovariectomy	After ovariectomy
Progesterone (ng/mL)	55.9 ± 7.2	14.2 ± 2.2*
Estradiol (pg/mL)	112.7 ± 10.8	41.1 ± 10.2*
Follicle-stimulating hormone (mIU/mL)	42.2 ± 4.0	257.3 ± 60.3*
Luteinizing hormone (mIU/mL)	1.3 ± 0.2	36.5 ± 10.4*

*Significant difference compared to before ovariectomy; Results are mean ± SEM (*n* = 7/group).

### 3.2 Effect of nitrate administration on heart function and SBP

**FIGURE 2:**
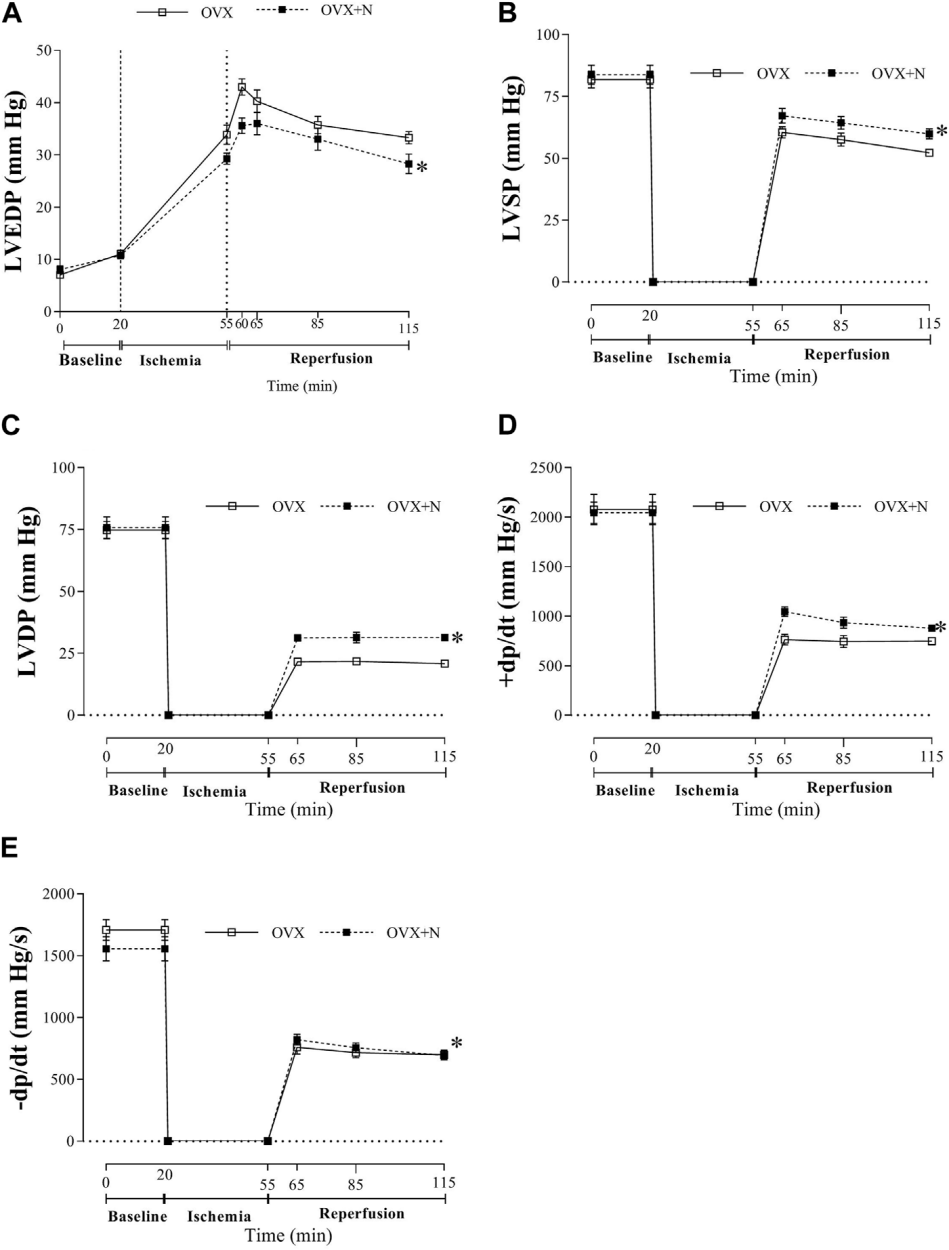
Tolerance to myocardial ischemia-reperfusion injury following nitrate administration in ovariectomized (OVX) rats as measured by left ventricular end-diastolic pressure (LVEDP, **(A)**, LV systolic pressure (LVSP, **(B)**, LV developed pressure (LVDP, **(C)**, peak rate of positive (+dp/dt, **(D)** and negative (-dp/dt, **(E)** changes in LV pressure. (*n* = 7/subgroup). OVX, ovariectomized; OVX + N, OVX + nitrate. * Significant difference compared to OVX rats.

Nitrate administration for 9 months to OVX rats did not affect baseline heart function [LVEDP (8.1 ± 0.7 vs 7.1 ± 0.4 mm Hg), LVSP (83.8 ± 3.9 vs 81.8 ± 3.3 mm Hg), LVDP (75.7 ± 4.3 vs 74.8 ± 3.4 mm Hg), +dp/dt (2048 ± 108 vs 2076 ± 152 mm Hg/s), and–dp/dt (1555 ± 98 vs 1708 ± 84 mm Hg/s)]. However, as shown in Figure 2, when subjected to ischemia, hearts from OVX + N rats showed decreased LVEDP (*p* < 0.001) and increased recovery of LVSP, LVDP, +dp/dt, and–dp/dt (all *p* < 0.001) during IR period. These results indicate that nitrate exerts protective effects against impaired heart function in OVX rats after IR but had no positive effect before IR.

In addition, nitrate administration decreased *in vivo* SBP (111 ± 6 vs 126 ± 3, *p* = 0.040) but did not affect *in vivo* HR (318 ± 19 vs 322 ± 14) in OVX rats.

### 3.3 Effect of nitrate administration on NO metabolites

**FIGURE 3:**
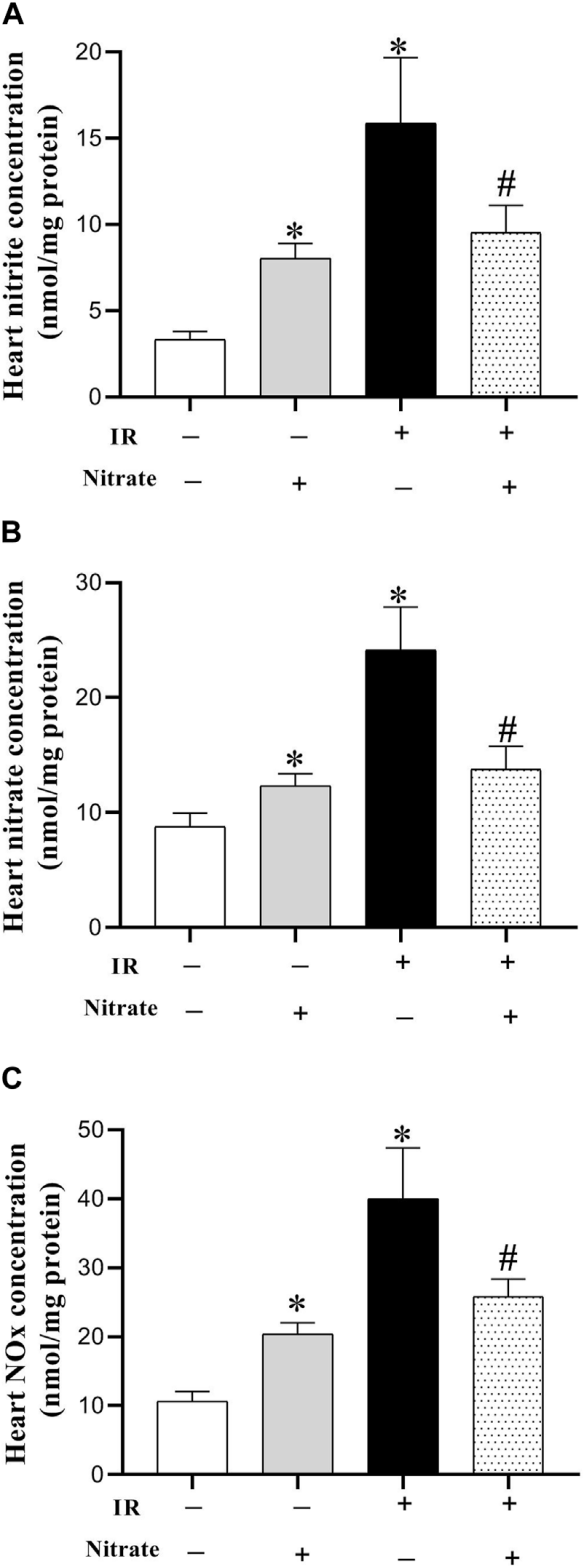
Concentration of nitrite **(A)**, nitrate **(B)**, and nitric oxide (NO) metabolites (NOx) **(C)**, at month nine in not-exposed (IR^−^) and exposed to ischemia-reperfusion (IR^+^) heart in ovariectomized rats. Results are mean ± SEM (*n* = 7/group). *, and ^#^ significant difference compared to IR^−^ and IR^+^ hearts from OVX rats, respectively.

Nitrate administration in OVX rats increased nitrite (142%, *p* < 0.001), nitrate (41%, *p* = 0.043), and NOx concentrations (91%, *p* < 0.001) in IR^−^ hearts. Exposed to IR increased nitrite (389%, *p* = 0.002), nitrate (175%, *p* < 0.001), and NOx (274%, *p* = 0.002) levels in OVX rats. Nitrate administration in OVX rats decreased nitrite (40%, *p* < 0.001), nitrate (43%, *p* = 0.027), and NOx (35%, *p* = 0.004) levels in IR^+^ hearts (Figure 3).

### 3.4 Effect of nitrate administration on mRNA expression of NOS enzymes and marker of apoptosis

**FIGURE 4:**
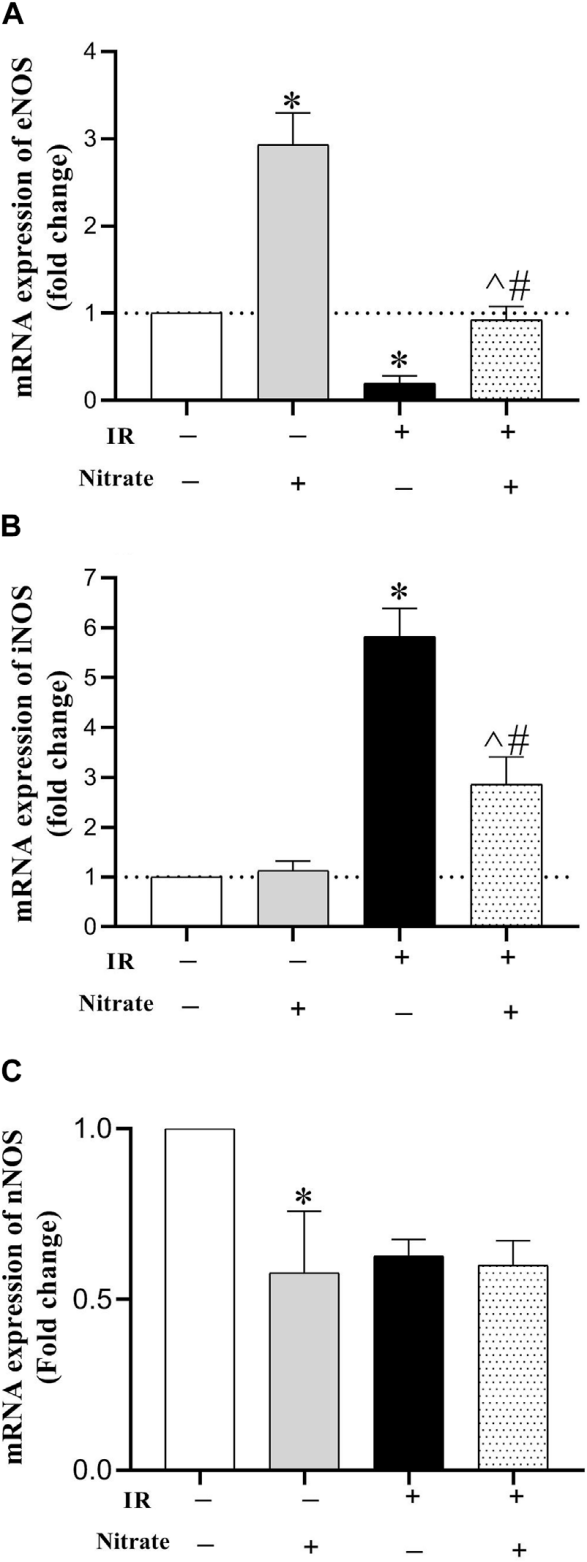
mRNA expression of the endothelial NO synthesis (eNOS) **(A)**, inducible NOS (iNOS) **(B)** and neural NOS **(C)** at month nine in not-exposed (IR^−^) and exposed to ischemia-reperfusion (IR^+^) heart in ovariectomized rats. (*n* = 7/group).*, ^#^, and ^ significant difference compared to IR^−^ and IR^+^ hearts from OVX rats and IR^−^ hearts from OVX + N rats, respectively.

As shown in Figure 4, nitrate administration to OVX rats increased eNOS (2.94-fold, *p* < 0.001), did not affect iNOS, and decreased nNOS (42%, *p* = 0.027) expressions in IR^−^ hearts. Exposed to IR in OVX rats, decreased eNOS (81%, *p* = 0.038), increased iNOS (5.82-fold, *p* < 0.001), and did not affect nNOS expressions. While nitrate administration to OVX rats increased eNOS (*p* = 0.004), decreased iNOS (*p* < 0.001), and did not affect nNOS mRNA expression in IR^+^ hearts; however, the eNOS expression remained lower by 18% (*p* = 0.077), and iNOS expression remained higher by 186% (*p* < 0.001) compared to IR^−^ hearts.

**FIGURE 5:**
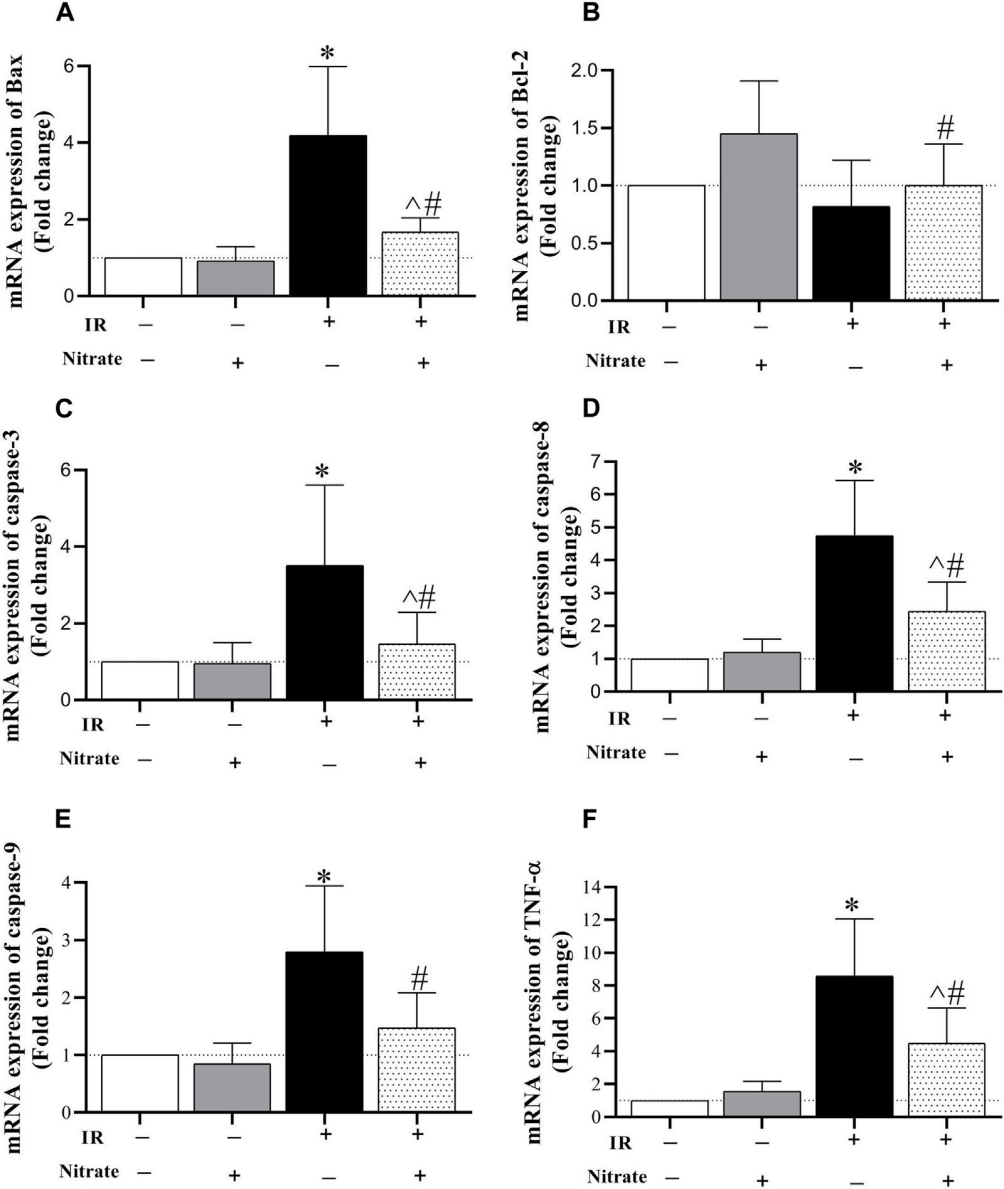
mRNA expression of the Bcl-2 associated X protein (Bax, **(A)**, B-cell lymphoma 2 (Bcl-2, **(B)**, caspase-3 **(C)**, caspase-8 **(D)**, caspase-9 **(E)**, and tumor necrosis factor-α (TNF-α, **(F)** expression at month nine in not-exposed (IR^−^) and exposed to ischemia-reperfusion (IR^+^) heart in ovariectomized rats. (*n* = 7/group).*, ^#^, and ^ significant difference compared to IR^−^ and IR^+^ hearts from OVX rats and IR^−^ hearts from OVX + N rats, respectively.

As shown in Figure 5, nitrate administration to OVX rats did not affect the expression of Bax, Bcl-2, caspase-3, caspase −8, caspase-9, and TNF-α in IR^−^ hearts. Exposed to IR in OVX rats increased Bax (319%, *p* < 0.001), caspase-3 (251%, *p* < 0.001), caspase-8 (375%, *p* < 0.001), caspase-9 (380%, *p* < 0.001), and TNF-α (759%, *p* < 0.001) expression. Nitrate administration decreased Bax (*p* < 0.001), caspase-3 (*p* < 0.001), caspase-8 (*p* = 0.003), caspase-9 (*p* = 0.003), and TNF-α (*p* = 0.011) expression, while increased Bcl-2 expression (*p* = 0.002) in IR^+^ hearts. However, the expression of Bax, caspase-3, caspase-8, and TNF-α remained higher by 67%, (*p* = 0.041), 47%, (*p* = 0.032), 144%, (*p* < 0.001) and 349% (*p* < 0.001) compared to IR^−^ hearts. These results indicate the anti-apoptotic effect of nitrate administration in IR^+^ hearts from OVX rats.

### 3.5 Effect of nitrate administration on infarct size levels

**FIGURE 6:**
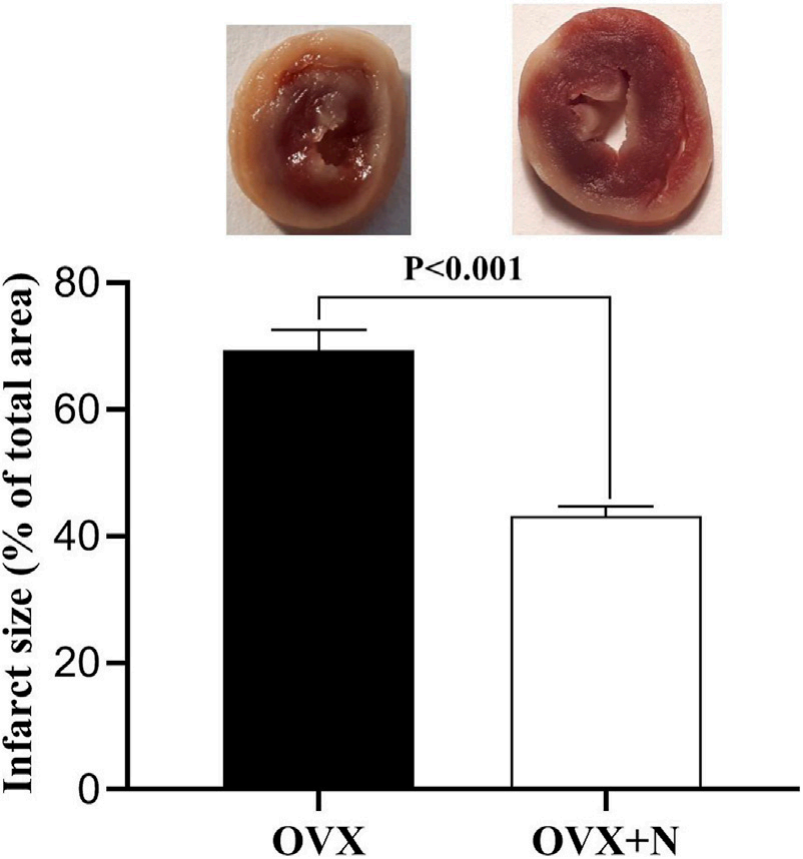
Levels of infarct size in the ischemia-exposed hearts from OVX and OVX + N rats. *n* = 7/subgroup. OVX, ovariectomized; OVX + N, OVX + nitrate.

Compared to IR^−^ hearts from OVX rats, nitrate administration decreased infarct size by 26.2% (*p* < 0.001) in IR^+^ hearts from OVX + N rats (Figure 6).

### 3.6 Effect of nitrate on oxidative stress indices in serum and heart tissue

Nitrate administration for 9 months increased serum TAC levels (*p* = 0.006) and CAT activity (*p* = 0.016) and decreased MDA levels (*p* = 0.022) at month 9. As shown in Table 3, nitrate administration for 9 months only increased CAT (*p* = 0.020) activity in IR^−^ hearts but increased TAC levels (*p* = 0.002), CAT activity (*p* < 0.001), and decreased MDA levels (*p* = 0.023) in IR^+^ hearts; that indicating inhibitory effects of nitrate against OVX-induced oxidative stress in circulation and heart tissue.

**TABLE 3:** Changes in serum and heart oxidants and antioxidants levels following 9 months of nitrate administration in ovariectomized (OVX) rats.

Animal group		
	OVX	OVX + N
**Serum**		
TAC (µM)	34.1 ± 9.3	95.0 ± 14.7^*^
CAT (U/L)	7.1 ± 0.9	12.3 ± 1.2^*^
MDA (µM)	10.2 ± 0.9	6.1 ± 0.7^*^
**Heart tissue before ischemia**		
TAC (mM)	1.3 ± 0.4	2.0 ± 0.4
CAT (U/L)	4.6 ± 0.8	9.1 ± 1.5^*^
MDA (µM)	2.6 ± 0.4	2.4 ± 0.3
**Heart tissue after ischemia**		
TAC (mM)	0.3 ± 0.1	1.4 ± 0.3^*^
CAT (U/L)	1.8 ± 0.2	8.6 ± 1.4^*^
MDA (µM)	5.7 ± 0.5	3.8 ± 0.5^*^

Data are mean ± SEM. *n* = 7/group; CAT, catalase; MDA, malondialdehyde; TAC, total antioxidant capacity.* Significant difference compared to OVX, rats.

## 4 Discussion

The findings of the present study show that long-term (9 months) low-dose (100 mg/L) nitrate preconditioning alleviated IR-induced myocardial injury in OVX rats that were confirmed by the higher recovery of CFI (decreased LVEDP and increased LVDP and ±dp/dt), and lower infarct size. The favorable effects of nitrate in OVX rats were associated with the eNOS upregulation before ischemia and the blunting of OVX-induced eNOS downregulation, iNOS upregulation, apoptosis, and oxidative stress in heart tissue after IR.

In the current study, in addition to the increase in body weight, serum concentrations of progesterone and estradiol decreased, while those of LH and FSH increased at month 0 (2 months after ovariectomy and before nitrate intervention), confirming the successful establishment of the OVX model (Yousefzadeh et al., 2020). The body weight-lowering effect of nitrate in OVX rats is attributed to its ability to mitigate OVX-induced adiposity (Yousefzadeh et al., 2022c), as evidenced by reduced adipocyte size and increased levels of brown adipose tissue (Yousefzadeh et al., 2022c; Yousefzadeh and Jeddi, 2022).

In this study, IR^+^ hearts from OVX rats showed higher recovery of CFI and lower infarct size following nitrate administration. Reduced CFI and increased infarct size in rats following 2 (Kaya and Agan, 2022), 4 (Ibañez et al., 2022), 5 (Ibañez et al., 2022), 6 (Garvin et al., 2017), 12 (Kumar et al., 2021), and 44 (Yousefzadeh and Jeddi, 2023) weeks of ovariectomy have previously been documented. Our findings align with previous studies (summarized in Table 4) showing reduced infarct size and improved post-ischemic cardiac function in healthy mice following short-term nitrate administration (7 days) at doses of 1000 mg/L (5 mg/day) (Bryan et al., 2007) and 43 mg/L (0.22 mg/day; equivalent to 10 g/L beetroot juice containing ∼0.7 mM nitrate) (Salloum et al., 2015). In addition, we previously reported the protective effects of long-term nitrate administration (100 mg/L, 3.5 mg/day) against myocardial IR injury in male diabetic rats (60 days) (Jeddi et al., 2016) and normal female rats (270 days) (Yassaghi et al., 2023b). Furthermore, nitrite administration (50 mg/L, 0.25 mg/day for 7 days) decreased infarct size in healthy and eNOS-deficient mice (Bryan et al., 2007; Bryan et al., 2008). Our data extend this effect to OVX rats and is in line with the results of a recent meta-analysis reported that nitrite/nitrate administration reduces myocardial infarct size by ∼15% in normal animals (Yassaghi et al., 2023a) and that nitrate is more effective if it is used as a preconditioning (Yassaghi et al., 2023a), as it was used in our study.

**TABLE 4:** Effect of nitrite/nitrate administration on myocardial ischemia-reperfusion injury in rodents.

Study	Animal	Condition	Sex	Intervention	Dose (mg/day)	Duration (day)	Response to myocardial IR injury	Type of ischemia
Bryan et al., 2007	Mouse	Healthy	NR	Nitrite	0.25	7	↑ Resistance	Regional
Bryan et al., 2008	Mouse	eNOS-deficient	NR	Nitrite	0.25	7	↑ Resistance	Regional
Bryan et al., 2007	Mouse	Healthy	NR	Nitrate	5	7	↑ Resistance	Regional
Salloum et al., 2015	Mouse	Healthy	Male	Nitrate	0.22	7	↑ Resistance	Regional
Jeddi et al., 2016	Rat	Diabetic	Male	Nitrate	3.5	60	↑ Resistance	Global
Yasaghi et al., 2023a	Rat	Healthy	Female	Nitrate	3.5	270	↑ Resistance	Global

eNOS, endothelial nitric oxide synthase.

NR, not reported.

In our study, the effect of nitrate against IR-induced myocardial injury in OVX animals was related to increased eNOS-derived NO both before ischemia and after IR, as well as decreased iNOS-derived NO after IR. Lower eNOS-derived NO (lower nitrate, nitrite, and NOx concentration and eNOS expression) before ischemia (Chou et al., 2010; Yousefzadeh and Jeddi, 2023) and higher iNOS-derived NO (higher nitrate, nitrite, and NOx concentration and higher iNOS expression) during IR (Sakanashi et al., 2013; Yousefzadeh and Jeddi, 2023) in the heart tissue have been reported in OVX rats. In line with our study, nitrate administration (7 days, 1000 mg/L (Bryan et al., 2007); 60 days, 100 mg/L (Jeddi et al., 2016); and 270 days, 100 mg/L (Yassaghi et al., 2023b)) increased eNOS-derived NO before ischemia and blunted decrease in eNOS and increased in iNOS expressions after IR in the heart tissue of diabetic male rats (Jeddi et al., 2016), normal female rats (Yassaghi et al., 2023b), and normal mice (Bryan et al., 2007). In normal heart tissue, eNOS produces about 80% of the heart’s NO, but during ischemia, iNOS is the predominant source of NO (Ghasemi and Jeddi, 2022). eNOS downregulation (Sharp et al., 2002) and iNOS overexpression (Di Napoli et al., 2001) exacerbate myocardial IR injury in animals; in contrast, eNOS overexpression (Jones et al., 2004) and iNOS suppression decrease myocardial IR injury (Sam et al., 2001) in animals.

Another potential mechanism underlying the favorable effects of nitrate against myocardial IR injury, observed in our study, is the reduction of circulating and myocardial oxidative stress before and after exposure to IR. Lower glutathione (GSH) levels and CAT activities and higher MDA and reactive oxygen species (ROS) levels have been reported in the heart tissue of OVX rats before and after exposure to IR (Tang et al., 2016; Kumar et al., 2021). Nitrate exerts antioxidant effects, as have been documented in the serum of OVX rats (Yang et al., 2017), the cardiovascular system of hypertensive rats (Amaral et al., 2019), rats with congestive heart failure (Donnarumma et al., 2016), and aged mice (Sindler et al., 2011). Nitrate inhibits the expression and activity of pro-oxidant enzymes and enhances the expression of antioxidant enzymes (Gao et al., 2015; Ghasemi et al., 2023), thereby exerting its antioxidant effects.

In the current study, nitrate administration reduced myocardial apoptosis in IR^+^ hearts from OVX rats, evidenced by the decreased mRNA expression of Bax, caspase-3, caspase-8, and caspase-9 as well as TNF-α. Activation of Fas receptor-dependent (TNF-α, Fas ligand, Fas death receptors, Fas-associated death domain, and caspase-8) and mitochondrion-dependent (Bax, Bax/Bcl-2 ratio, cytochrome c, and caspase-9) apoptotic pathways have been reported in the hearts of OVX rats (Huang et al., 2016; Wu et al., 2022). Consistent with our findings, beetroot juice (150 and 300 mg/kg for 28 days) (Raish et al., 2019) and nitrite/nitrate administration (60-min nitrite infusion 0.20 μmol/min/kg (Gonzalez et al., 2008) or 50 μL containing 1.2–1,920 nmol sodium nitrite or sodium nitrate (Duranski et al., 2005)) decreased apoptosis in IR^+^ hearts of control rats. Bryan et al. proposed that nitrite, derived from dietary nitrate, plays two critical roles in heart tissue during IR; it acts as a non-enzymatic NO source during ischemia and also reacts with thiol groups to form S-nitrosothiols, which prevent protein/lipid oxidation (Bryan et al., 2007) and apoptosis (Mannick et al., 1999). NO-derived nitrate mitigates apoptosis by inhibiting caspase activity, preventing mitochondrial membrane potential loss, and reducing cytochrome c release (Sellers et al., 2012). Nitrite S-nitrosates complex I of the mitochondrial electron transport chain, decreasing electron flow and ROS formation during reperfusion (Shiva et al., 2007), which inhibits the opening of the mitochondrial permeability transition pore, reducing cytochrome c release and limiting apoptosis (Gonzalez et al., 2008).

Nitrate is reduced to nitrite and then to NO; while nitrite itself can directly bind to ERα (Veselik et al., 2008), NO primarily S-nitrosylates the receptor (Garbán et al., 2005), potentially enhancing its activity. Thus, it can be speculated that nitrate has estradiol-like effects in cardiac tissue, including decreasing oxidative stress (Sedeek et al., 2013; Vrtačnik et al., 2014), iNOS activity (Chang et al., 2002), endogenous eNOS inhibitor (Monsalve et al., 2007), and increasing eNOS expression and activity (Wassmann et al., 2002; Stirone et al., 2003). The effectiveness of estrogen therapy for women with surgical menopause has been questioned because of its side effects (Marjoribanks et al., 2012; Sarrel et al., 2016; Garg et al., 2020), and inorganic nitrate, which can be easily obtained through vegetable consumption, may potentially be an alternative.

This study has some strengths. First, the dose of nitrate used in rats (100 mg/L equals 11 mg/kg/day) is translated to 1.8 mg/kg in humans, which is in the range of the median nitrate intake in humans (1.28–2.14 mg/kg/day) (Reagan-Shaw et al., 2008; Babateen et al., 2018). This dose is found in green leafy vegetables and has beneficial effects against cardiometabolic diseases (Lundberg et al., 2018; Kapil et al., 2020). Second, the OVX rat model used in the present study mimics the menopause-induced changes observed in women after surgical menopause (Yousefzadeh et al., 2020). Finally, given that a living day in rats is roughly equivalent to 26 days in humans (Quinn, 2005), a 9-month nitrate intervention could be considered a long-term intervention in humans. This study has some limitations; first, we did not use pharmacological interventions to directly assess the causal roles of NOS enzymes and ERα in the observed cardioprotective effects of nitrate. However, considering the protective effect of eNOS-derived NO (Sharp et al., 2002) and the detrimental effect of iNOS-derived NO (Parlakpinar et al., 2005; Li et al., 2006) following myocardial IR injury, it can be assumed that the protective effects of nitrate against myocardial IR injuries in OVX rats could be blunted after acute inhibition of eNOS or enhances after acute inhibition of iNOS. Second, the study did not measure heart histology to investigate the potential protective effects of nitrate against OVX-induced structural damage in heart tissue. Third, this study used a global ischemia model, commonly used in the Langendorff-perfused heart model. While the regional ischemia model may be clinically more relevant (Kim et al., 2012), the global ischemia model offers simplicity and reproducibility of results (Bibli et al., 2012). However, in the global ischemia model, the direct measurement of infarcted and non-infarcted areas is impractical or inaccurate. Fourth, the study did not measure coronary flow, an indicator of isolated heart function, such as the heart’s ability to maintain cardiac output. Finally, the rats were followed for 9 months after nitrate treatment, and the response to myocardial IR injury was measured at month 9. Longer or shorter follow-up periods may yield different results.

## 5 Conclusion

This study demonstrates that preconditioning with low-dose (100 mg/L) of nitrate for a long term (9 months) mitigates IR-induced myocardial injury in OVX animals. The protective effects of nitrate were evidenced by the enhanced recovery of cardiac function (decreased LVEDP and increased LVDP, ±dp/dt) and reduced infarct size. These favorable outcomes are associated with nitrate’s ability to upregulate eNOS levels before IR and to prevent OVX-induced eNOS downregulation, iNOS upregulation, apoptosis, and oxidative stress in heart tissue after IR.

One possible implication of our findings that needs to be tested in human studies is that regular intake of nitrite/nitrate-rich foods may offer protection against myocardial IR injury in women after surgical menopause. Estimates indicate that consuming nitrite-containing foods, such as leafy green vegetables and fruits, can decrease the risk of IHD (Bryan, 2017). This notion is supported by studies showing a reduced risk of IHD mortality in vegetarians, who often consume higher amounts of nitrite/nitrate-rich vegetables (average follow-up of 10.68 years), compared to non-vegetarians (Jabri et al., 2021). Furthermore, increasing daily fruit and vegetable intake has been linked to a decreased risk of coronary heart disease by 4% (Joshipura et al., 2001). Regular consumption of nitrite/nitrate-rich foods can compensate for disturbances in endogenous NO synthesis (Lundberg et al., 2006), making this a potential protective strategy for individuals at risk of CVD (Bryan, 2006). This risk is particularly elevated in women after surgical menopause, an NO-deficient state linked to a 50% higher risk of IHD compared to natural menopause (Muka et al., 2016). In addition, some studies proposed reclassifying nitrite as a dietary mineral due to its natural presence in some foods (Bryan, 2017). They also suggest exploring potential food fortification with nitrate and nitrite (Bryan and Ivy, 2015) as strategies worth further investigation. Therefore, modest dietary changes to include nitrite/nitrate-rich foods could be particularly beneficial for women who have undergone surgical menopause and are at a higher risk of myocardial IR.

## Data Availability

The original contributions presented in the study are included in the article/Supplementary material, further inquiries can be directed to the corresponding authors.
